# Supplementary material for: The interactome of intact mitochondria by cross-linking mass spectrometry provides evidence for coexisting respiratory supercomplexes
Source: Mol Cell Proteomics. 2017 Dec 8;17(2):216–32. doi: 10.1074/mcp.RA117.000470 (PMC5795388; doi:10.1074/mcp.RA117.000470)
Supplement: Supplemental Data [file supp_RA117.000470_133922_0_supp_23978_fzffwf.zip › spectra_annotation/mito_spectra_annotation/213-1-2-1-7-1.pdf]

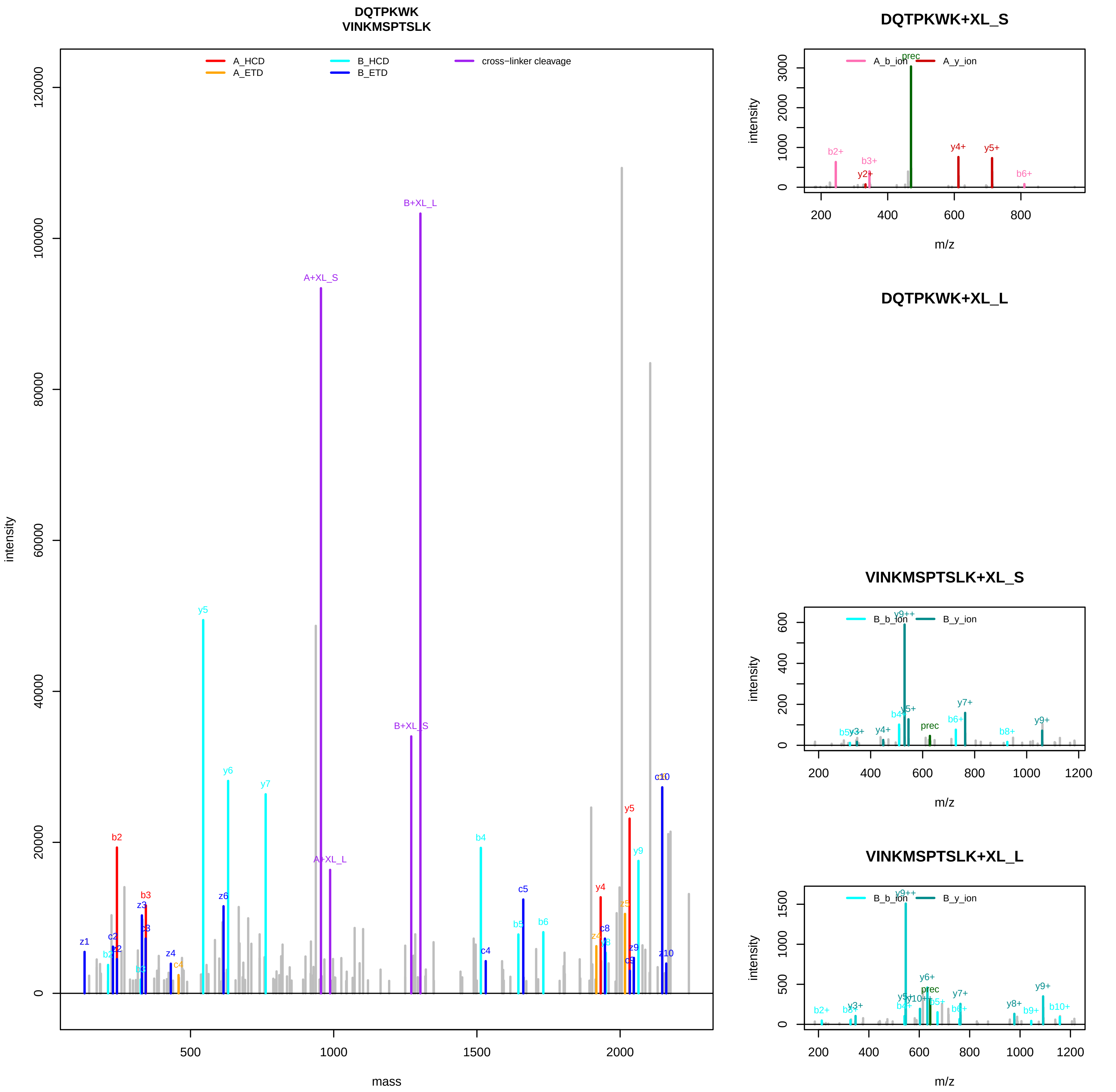
DQTPKWK
VINKMSPTSLK
A_HCD
A_ETD
B_HCD
B_ETD
cross−linker cleavage
intensity
mass
0
20000
40000
60000
80000
100000
120000
500
1000
1500
2000
A+XL_S
B+XL_L
B+XL_S
A+XL_L
y5
y6
y7
b2
b3
z1
c2
z2
z3
c3
z4
c4
z6
b4
c4
c5
b5
b6
y4
y5
z5
c8
y9
c9
z9
c10
z10
DQTPKWK+XL_S
A_b_ion
A_y_ion
prec
b2+
b3+
y2+
y4+
y5+
b6+
m/z
0
1000
2000
3000
200
400
600
800
DQTPKWK+XL_L
VINKMSPTSLK+XL_S
B_b_ion
B_y_ion
y9++
b4+
y5+
y4+
b5+
y3+
prec
b6+
y7+
b8+
y9+
0
200
400
600
200
400
600
800
1000
1200
VINKMSPTSLK+XL_L
y9++
y6+
prec
y10++
b5+
y7+
b2+
b3+
y3+
b4+
y8+
b9+
y9+
b10+
b6+
0
500
1000
1500
